# Risk for Avian Influenza Virus Exposure at Human–Wildlife Interface

**DOI:** 10.3201/eid1407.080066

**Published:** 2008-07

**Authors:** Jennifer Siembieda, Christine K. Johnson, Walter Boyce, Christian Sandrock, Carol Cardona

**Affiliations:** * University of California, Davis, School of Veterinary Medicine, Davis, California, USA; † University of California, Davis, School of Medicine, Sacramento, California, USA

**Keywords:** Avian influenza, influenza A, wildlife, dispatch

## Abstract

To assess risk for human exposure to avian influenza viruses (AIV), we sampled California wild birds and marine mammals during October 2005–August 2007and estimated human–wildlife contact. Waterfowl hunters were 8 times more likely to have contact with AIV-infected wildlife than were persons with casual or occupational exposures (p<0.0001).

The emergence of highly pathogenic avian influenza virus (AIV) (H5N1) in domestic poultry in Asia with spillover infections in humans has raised concerns about the potential for a human pandemic (*1*). Although subtype H5N1 is the most well-known infecting strain, evidence of direct bird-to-human transmission has been documented for several other AIV subtypes (*2*).

Little is known about the types of exposure that result in human infections, especially with AIV being transmitted from wild birds and animals because only a few cases of transmission to humans have been documented (*3*–*5*). Overall, the types of exposures associated with the transmission of AIV to humans have been ingestion, inhalation of aerosolized virus, or direct contact through mucous membranes (2,*4*). The probability of infection with AIV varies with the activity and depends on the contact type (duration and route) and dose. Contacts for the general public are likely short and indirect, often occurring through outdoor activities, such as hiking, picnicking, or feeding birds. Contact for waterfowl hunters is especially intense and direct during bird-cleaning activities. Biologists and workers at wildlife hospitals have frequent and direct contact with wild birds and mammals. Biologists trap apparently healthy free-ranging animals and perform field necropsies, and rehabilitation workers handle sick and injured wild animals. In this study, we tested wild birds and marine mammals for AIV to determine the exposure risks associated with specific casual, recreational, and occupational activities that result in contact with wildlife.

## The Study

Human risk categories were created based on a typical contact type with wildlife: 1) casual (the general public), 2) recreational (waterfowl hunters), and 3) occupational (wildlife biologists, wildlife hospital workers, and veterinarians). Frequency of contact with AIV was estimated for each risk group by evaluating the prevalence of AIV among animals sampled opportunistically in each category. Surveillance for AIV was conducted from October 2005 through August 2007.

For casual contact, wild bird species (mostly periurban passerines such as sparrows, finches, and crows) were sampled to reflect typical daily exposures for the public (Figure). For recreational contact, birds were assessed by sampling hunter-killed waterfowl (mostly mallards, northern shovelers, gadwalls, green-winged teals, northern pintails, and American widgeons) at check stations in the Sacramento National Wildlife Refuge. For occupational contact, wild birds (seabirds, wading birds, waterfowl, raptors, and passerines) and marine mammals (seals and sea lions) admitted to 3 northern California wildlife hospitals were sampled. Cloacal samples were taken from birds and nasal and rectal samples from marine mammals with rayon-tipped swabs (MicroPur; PurFybr, Inc., Munster, IN, USA). Birds in recovery also had oropharyngeal samples taken. Swab samples were placed in viral transport media, transported within 24 hours from the site of collection to the University of California, Davis, in a cooler with ice packs and then transferred to a –70°C freezer for storage. A total of 9,157 samples were tested for AIV. Of these, 2,346 were screened by virus isolation in embryonating chicken eggs (*6*,*7*), and 6,811 were screened by real-time reverse transcription–PCR (RT-PCR) (*7*). All positive samples were tested for Eurasian H5 viruses (*8*).

**Figure:**
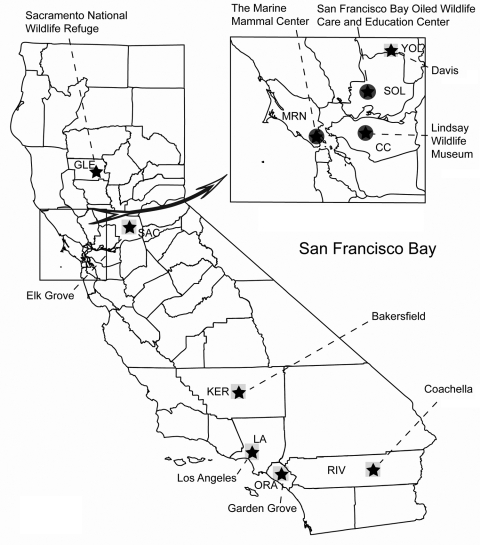
Map of California displaying sample collection sites for avian influenza testing, fall 2005–summer 2007. The casual risk category is represented by a square, recreational risk category by a star, and occupational risk category by a circle. Counties are abbreviated as follows: CC, Contra Costa; GLE, Glenn; KER, Kern; LA, Los Angeles; MRN, Marin; ORA, Orange; RIV, Riverside; SAC, Sacramento; SOL, Solano; YOL, Yolo.

The prevalence of AIV in each group was low (ranged 0.1%–0.9%) (Table), and no samples were positive for Eurasian H5. We found that risk of contact with AIV-infected wildlife was 8 times higher for the recreational group compared to either the occupational or the casual group (p<0.0001; EpiInfo, Centers for Disease Control and Prevention, Atlanta, GA, USA).

**Table:** Prevalence of avian influenza viruses in California wild birds and marine mammals, October 2005–August 2007, categorized by exposure risk category

Exposure risk group	No. positive (%)	No. tested	Species (no. positive)
Casual	8 (0.2)	4,757	Finch (3), sparrow (2), cowbird (1), quail (2)
Recreational	20 (0.9)	2,346	Duck (19), goose (1)
Occupational	2 (0.1)	2,054	Seabird (1), egret (1)
Total	30 (0.3)	9,157	

## Conclusions

We did not detect AIV (H5N1) in California during October 2005–August 2007 nor did other surveillance efforts in the United States (*9*). We did detect other AIVs, although at a low prevalence (<1%). The prevalence of AIV in California wildlife was substantially lower than the prevalence reported in Alaskan wildlife in the same flyway (*10*). AIV prevalence may decrease with latitude (*11*), or this opportunistic sample design may have resulted in testing of species with a natural low prevalence. Although overall prevalence was low, it was highest in the recreational category and, coupled with the directness and intensity of the contacts especially during bird cleaning, this group would be expected to have the highest risk for infection. However, emergence or introduction of a virus that causes disease in wild birds or animals would likely result in a disproportional shift in prevalence of infection in wildlife brought to rehabilitation hospitals, thus making occupational contact more risky. As a recent example, 1 stork and 2 buzzards that were infected with AIV (H5N1) were brought to a wildlife hospital in Poland, which potentially exposed staff (*12*).

Novel transmission pathways are possible in places like wildlife hospitals because wild species that do not meet in nature are brought into close and extended contact with each other and humans. For example, marine mammals are susceptible to infection with AIV (*4*) and human influenza viruses (*13*) and have been documented as intermediate hosts (*4*). Other species may also be intermediate hosts for AIV, although they have not been identified. Those working in wildlife occupations should be encouraged to wear personal protective equipment when handling wildlife because of the types of contacts they can have and the potential for viruses to emerge in this setting. Similarly, personal protection should be recommended for waterfowl hunters because of the relatively higher prevalence of AIV in the birds with which they have contact.

We assessed the risk for human exposure to AIV by opportunistically sampling wildlife at the human–wild animal interface. A better measure of human risk would be to directly assess human exposure by testing for antibodies to all AIV subtypes that could occur in nature. Although it is not practical to simultaneously test for 144 virus subtypes, 2 serologic studies of persons exposed to wildlife showed antibodies to a limited number of AIVs (*3*,*14*). Since these exposures did not cause discernable illness, diagnosis based on clinical signs would likely underestimate infection.

Although our methods enabled us to compare exposure risk among different groups, the testing methods we used likely did not estimate the true AIV prevalence in wildlife. The real-time RT-PCR used in this study and in national surveillance efforts (*7*) has not been validated in wildlife (*10*), nor has virus isolation in embryonating chicken eggs, and it may be that neither method is perfect in detecting AIV in species that are only distantly related to chickens (*15*). Improved diagnostic methods are needed to assess AIV infections in wildlife species, and close monitoring of persons with the highest level of exposure to AIV is a necessary component of an early warning system to detect transmission from animals to humans.
